# Disparities in Access to Trauma Care in Sub-Saharan Africa: a Narrative Review

**DOI:** 10.1007/s40719-022-00229-1

**Published:** 2022-06-06

**Authors:** Barnabas Alayande, Kathryn M. Chu, Desmond T. Jumbam, Oche Emmanuel Kimto, Gambo Musa Danladi, Alliance Niyukuri, Geoffrey A. Anderson, Deena El-Gabri, Elizabeth Miranda, Mulat Taye, Ngyal Tertong, Tolgou Yempabe, Faustin Ntirenganya, Jean Claude Byiringiro, Augustine Z. Sule, Olive C. Kobusingye, Abebe Bekele, Robert R. Riviello

**Affiliations:** 1 Center for Equity in Global Surgery, University of Global Health Equity, Kigali, Rwanda; 2 Program in Global Surgery and Social Change, Harvard Medical School, Boston, MA USA; 3 Centre for Global Surgery, Department of Global Health, Faculty of Medicine and Health Sciences Stellenbosch University, Cape Town, South Africa; 4 Department of Policy and Advocacy, Operation Smile, Accra, Ghana; 5 Surgical Equity and Research Centre, Jos, Nigeria; 6 Hope Africa University, Bujumbura, Burundi; 7 Mercy Surgeons-Burundi, Research Department, Bujumbura, Burundi; 8 Mercy James Center for Paediatric Surgery and Intensive Care-Blantyre, Blantyre, Malawi; 9 Department of Surgery, Brigham and Women’s Hospital, Boston, MA USA; 10 Center for Surgery and Public Health, Brigham and Women’s Hospital, Boston, MA USA; 11 School of Medicine, Addis Ababa University, Addis Ababa, Ethiopia; 12 International Fellow, Paediatric Orthopaedic Surgery Department of Orthopaedics, Sheffield Children’s Hospital, Sheffield, UK; 13 Orthopaedic and Trauma Unit, Department of Surgery, Tamale Teaching Hospital, Tamale, Ghana; 14 University Teaching Hospital of Kigali, Kigali, Rwanda; 15 Department of Surgery, School of Medicine and Pharmacy, College of Medicine and Health Sciences, University of Rwanda, Kigali, Rwanda; 16 NIHR Research Hub On Global Surgery, University of Rwanda, Kigali, Rwanda; 17 School of Medicine and Pharmacy, College of Medicine and Health Sciences, University of Rwanda, Kigali, Rwanda; 18 Jos University Teaching Hospital, Jos, Nigeria; 19 Makerere University School of Public Health, Kampala, Uganda; 20 George Institute for Global Health, Sydney, Australia; 21 Department of Global Health and Social Medicine, Harvard Medical School, Boston, MA USA

**Keywords:** Trauma, Injury, Access, Disparity, Sub-Saharan Africa

## Abstract

**Purpose of Review:**

Sub-Saharan Africa is a diverse context with a large burden of injury and trauma-related deaths. Relative to high-income contexts, most of the region is less mature in prehospital and facility-based trauma care, education and training, and trauma care quality assurance. The 2030 Agenda for Sustainable Development recognizes rising inequalities, both within and between countries as a deterrent to growth and development. While disparities in access to trauma care between the region and HICs are more commonly described, internal disparities are equally concerning. We performed a narrative review of internal disparities in trauma care access using a previously described conceptual model.

**Recent Findings:**

A broad PubMed and EMBASE search from 2010 to 2021 restricted to 48 sub-Saharan African countries was performed. Records focused on disparities in access to trauma care were identified and mapped to de Jager’s four component framework. Search findings, input from contextual experts, comparisons based on other related research, and disaggregation of data helped inform the narrative. Only 21 studies were identified by formal search, with most focused on urban versus rural disparities in geographical access to trauma care. An additional 6 records were identified through citation searches and experts. Disparity in access to trauma care providers, detection of indications for trauma surgery, progression to trauma surgery, and quality care provision were thematically analyzed. No specific data on disparities in access to injury care for all four domains was available for more than half of the countries. From available data, socioeconomic status, geographical location, insurance, gender, and age were recognized disparity domains. South Africa has the most mature trauma systems. Across the region, high quality trauma care access is skewed towards the urban, insured, higher socioeconomic class adult. District hospitals are more poorly equipped and manned, and dedicated trauma centers, blood banks, and intensive care facilities are largely located within cities and in southern Africa. The largest geographical gaps in trauma care are presumably in central Africa, francophone West Africa, and conflict regions of East Africa. Disparities in trauma training opportunities, public–private disparities in provider availability, injury care provider migration, and several other factors contribute to this inequity. National trauma registries will play a role in internal inequity monitoring, and deliberate development implementation of National Surgical, Obstetrics, and Anesthesia plans will help address disparities. Human, systemic, and historical factors supporting these disparities including implicit and explicit bias must be clearly identified and addressed. Systems approaches, strategic trauma policy frameworks, and global and regional coalitions, as modelled by the Global Alliance for Care of the Injured and the Bellagio group, are key. Inequity in access can be reduced by prehospital initiatives, as used in Ghana, and community-based insurance, as modelled by Rwanda.

**Summary:**

Sub-Saharan African countries have underdeveloped trauma systems. Consistent in the narrative is the rural-urban disparity in trauma care access and the disadvantage of the poor. Further research is needed in view of data disparity. Recognition of these disparities should drive creative equitable solutions and focused interventions, partnerships, accompaniment, and action.

**Supplementary Information:**

The online version contains supplementary material available at 10.1007/s40719-022-00229-1.

## Introduction

“If it is true that surgery is the neglected stepchild of global health, does it follow that there are no surgical services available in the poor world? The truth is even more unpleasant: within poor countries, surgical services are concentrated almost wholly in cities and reserved largely for those who can pay for them.” ~ Paul Farmer and Jim Kim [1]

Globally, injury is responsible for more mortality than HIV/AIDS, malaria, and tuberculosis combined [2]. About 4.4 million people die from injury each year with over 90% of injury deaths occurring in low- and middle-income countries [3, 4]. From the ages of 10 to 49 years, road injuries specifically constitute the largest burden of disease and contribute 4.6 to 7.7% of all disability-adjusted life years, with sub-Saharan Africa (SSA) leading the incidence of road traffic injury-related deaths [3]. Over a third of these injury deaths can be prevented by access to adequate trauma care [5]. The consequence of lack of equitable access to trauma care is avoidable mortality and morbidity and loss of economic productivity for disadvantaged populations with perpetuation of a cycle of social and economic inequity [6, 7].

Trauma care is a significant consideration in emergency and essential surgical care [8, 9]. Based on the World Health Organization (WHO) Trauma System Maturity Index, most of SSA is less mature, relative to other regions, in prehospital trauma care, facility-based trauma care, education and training, and trauma care quality assurance, with only South Africa hosting a more advanced (WHO level III) trauma system [10]. Five billion/people globally lack access to surgical care and an overwhelming proportion of these reside in SSA [11••].

Multiple views of disparities are possible in the SSA context. These include disparities between SSA and the rest of the world, between subregions, between countries, and within countries. The 2030 Agenda for Sustainable Development recognizes rising inequalities, both within and between countries both as an impediment to growth and development and a violation of shared norms, values, and fairness [12]. This review will be limited to disparities existing within the region. The contrast in surgical equity between high-income countries (HICs) and African LMICs looms large [11••], but the internal inequities within and among SSA countries are equally concerning. In contrast to abundant HIC literature, specifically coming from the USA addressing internal disparities in surgical access and outcomes, [13] little attention has been paid to internal disparities within the LMIC sub-Saharan arena. In 2019, Ethiopia, Uganda, Zimbabwe, and South Africa had the highest proportions of death by injury in the region [14]. SSA is far from a homogeneous geographical or sociopolitical entity. Consisting of 46 nations on 17% of the world’s land area, and home to 1.1 billion people (see Table 1), this scope and range reflexively lend itself to disparities. Poverty is widespread, and in the presence of a large amount of the world’s natural resources, the region produces less than 2% of global gross domestic product [15] across its middle- and low-income World Bank categories (Table 1). Given that all of SSA is not developing at the same pace, focus on identifying and mitigating health and healthcare disparities should be prioritized. This review will guide equitable engagement by policymakers, advocates, surgical colleges, donors and volunteers, and organizations whose work in trauma care straddles SSA.

**Table 1:** Socio-demographics of sub-Saharan Africa

	Country	World Bank designation^a^	Population estimates^b^	Male:female ratio^c^	Rural: urban ratio^c^	Medical doctor density (per 10,000) ^c^	GDP per capita (current USD)^d^	Highest % GDP on health^e^	Lowest % GDP on health^e^	Representative ethnic majorities (≥ 10 million) ^f, g^	Representative ethnic minorities^f, g^
Sub-Saharan Africa			1,106,957,895				1,483.8	5.1%			
Africa Union Region											
West Africa			412,453,951	1:0.98	1.09:1			16.1%, Sierra Leone	2.5%,Benin	Akan, Fulani, Hausa, Igbo, Yoruba	Ogoni, Tuareg, Arabs
	Guinea-Bissau	LIC	1,967,998	1:1.02	1:0.93	1.3	727.5				
	The Gambia	LIC	2,416,664	1:1.03	1:1.67	1.1	787.0				
	Cape Verde	LMIC	555,988	1:1.02	1:1.99	7.8	3,064.3				
	Liberia	LIC	5,057,677	1:0.99	1:1.09	0.4	583.3				
	Sierra Leone	LIC	7,976,985	1:1.05	1:0.75	0.7	484.5				
	Togo	LIC	8,278,737	1:1.02	1:0.75	0.8	915.0				
	Guinea	LIC	13,132,792	1:0.98	1:0.58	0.8	1,194.0				
	Niger	LIC	24,206,636	1:0.99	1:0.19	0.4	565.1				
	Benin	LMIC	12,123,198	1:1.03	1:0.94	0.7	1,291.0				
	Burkina Faso	LIC	20,903,278	1:1.01	1:0.44	0.9	830.9				
	Mali	LIC	20,250,834	1:1.00	1:0.78	1.3	858.9				
	Senegal	LMIC	16,743,930	1:1.02	1:0.93	0.9	1,487.8				
	Côte d'Ivoire	LMIC	26,378,275	1:0.96	1:30.68	1.6	2,325.7				
	Ghana	LMIC	31,072,945	1:0.97	1:1.34	1.1	2,328.5				
	Nigeria	LMIC	206,139,587	1:0.97	1:1.08	3.8	2,097.1				
Central Africa			179,595,134	1:1.01	0.98:1			The Central African Republic 11%	Republic of Congo 2.1%	Chewa, Hutu, Kanuri, Kongo, Luba, Mongo	Gbaya,Banda, Mandjia, sara, Mboun, M'baka, Yakoma
	Central African Republic	LIC	4,829,764	1:1.03	1:1.09	0.7	476.9				
	São Tomé and Príncipe	LMIC	219,161	1:1.02	1:0.75	3.2	2,157.8				
	Republic of Congo	LMIC	5,518,092	1:0.99	1:0.75	1.1	1,972.5				
	Equatorial Guinea	UMIC	1,402,985	1:0.95	1:0.58	4.0	7,143.2				
	Chad	LIC	16,425,859	1:1.01	1:0.19	0.5	614.5				
	Gabon	UMIC	2,225,728	1:0.99	1:0.94	6.8	7,005.9				
	Cameroon	LMIC	26,545,864	1:1.00	1:0.44	0.9	1,499.4				
	Democratic Republic of the Congo	LIC	89,561,404	1:1.01	1:0.78	0.9	556.8				
	Angola	LMIC	32,866,268	1:1.02	1:0.93	2.2	1,895.8				
Eastern Africa			445,671,871	1:2.02	2.36:1			Malawi 9.3%	Tanzania 3.6%	Habesha, Amhara, Oromo, Bantu, Somali	Batwa (Twa), Saho, Maasai
	Seychelles	HIC	98,462	1:0.96	1:1.36	2.5	11,425.1				
	Comoros	LMIC	869,595	1:0.99	1:0.42	1.7	1,402.6				
	Eritrea	LIC	3,213,969	1:1.03	1:1.00	0.8	642.5				
	Burundi	LIC	11,890,781	1:1.04	1:0.16	1.0	274.0				
	Djibouti	LMIC	988,002	1:0.99	1:3.56	2.2					
	Somalia	LIC	15,893,219	1:1.02	1:0.86	0.2	309.4				
	Malawi	LIC	19,129,955	1:0.99	1:0.21	0.4	625.3				
	Rwanda	LIC	12,952,209	1:1.04	1:0.21	1.2	797.9				
	Mauritius	LIC	1,265,740	1:1.03	1:0.69	25.3	1,672.9				
	Zimbabwe	LMIC	14,862,927	1:1.03	1:0.48	2.1	1,128.2				
	Madagascar	LIC	27,691,019	1:1.04	1:0.63	1.8	495.5				
	Mozambique	LIC	312,554,35	1:1.05	1:0.59	0.9	448.6				
	Zambia	LIC	18,383,956	1:0.99	1:0.81	0.9	1,050.9				
	Tanzania	LIC	59,734,213	1:1.0	1:0.54	0.3	1,076.5				
	Uganda	LIC	45,741,000	1:1.10	1:0.33	1.7	817.0				
	South Sudan	LMIC	11,193,729	1:0.98	1:0.25	2.6	1,119.7				
	Kenya	LIC	53,771,300	1:1	1:0.39	1.6	1,838.2				
	Ethiopia	LIC	114,963,583				936.3				
	Sudan	LMIC	43,849,269				595.5				
Southern Africa		LMIC	67,503,635	1:1.02	0.5:1			Lesotho 9.3	Botswana 5.8	Zulu	San, Himba, Herero, Bemba
	Lesotho	LMIC	2,142,252	1:1.03	1:0.41	0.7	861.0				
	Eswatini	LMIC	1,160,164	1:1.04	1:0.32	0.9	3,415.5				
	Namibia	UMIC	2,540,916	1:1.01	1:1.08	5.9	4,211.1				
	Botswana	LIC	2,351,625	1:0.98	1:2.43	2.9	6,711.0				
	South Africa	LMIC	59,308,690	1:1.02	1:2.06	7.9					

^a^World Bank country and lending groups for 2022 fiscal year. Low-income economies (LICs) have a gross national income per capita of $1,045 or less in 2020; lower middle-income economies (LMICs) have a GNI per capita between $1,046 and $4,095, upper middle-income economies (UMICs) are those with a GNI per capita between $4,096 and $12,695, high-income economies (HICs) have GNI per capita of $12,696 or more. https://datahelpdesk.worldbank.org/knowledgebase/articles/906519-world-bank-country-and-lending-groups

^b^Data from the World Bank (2020), https://data.worldbank.org/indicator/SP.POP.TOTL?locations=ZG

^c^Data from the World Bank (2020), https://data.worldbank.org/indicator/NY.GDP.PCAP.CD?locations=ZG

^d^Data from the World Bank (2020) https://data.worldbank.org/

^e^2018 data from the World Bank https://data.worldbank.org/indicator/SH.XPD.CHEX.GD.ZS?locations=ZG

^f^Ethnic groups in Africa. https://resources.saylor.org/wwwresources/archived/site/wp-content/uploads/2011/04/Ethnic-groups-in-Africa.pdf

^g^Groups identified may not be exhaustive

### Defining Access

Access to trauma care is more complex than the unidimensional measure of the geographical density of operating theatres potentially capable of trauma surgery. Available, accessible, safe, and timely trauma surgical interventions define access [11••]. It is multidimensional and can be viewed through a framework of geography, time, structure, human and material resources, finance, and politics. Access encompasses the process of entering into and staying in the trauma care system, alongside quality of care [11••, 11]. Specialist surgical workforce density, surgical volume, perioperative mortality, protection against impoverishing, and catastrophic expenditure are viable adaptable trauma care indicators [11••].

### Defining Trauma Care Disparities

Not all differences in trauma care or outcomes are disparate. Concisely put, health differences that are avoidable, unnecessary, and unjust are true health disparities or inequities [18]. Once these differences systematically and negatively affect less privileged groups, they can be categorized as disparities. “A health disparity is defined as a particular type of health difference that is closely linked with social, economic, and/or environmental disadvantage. Health disparities adversely affect groups of people who have systematically experienced greater obstacles to health based on their racial or ethnic group; religion; socioeconomic status; gender; age; mental health; cognitive, sensory, or physical disability; sexual orientation or gender identity; geographic location; or other characteristics historically linked to discrimination or exclusion” [19]. It must be noted that the term “health disparities” is most commonly used within the USA, in contrast to the terms “health inequity” or “health inequality” more commonly understood outside the USA to refer to the similar concepts [20]. In Western HICs, the predominant demographic domain associated with disparity is race. The international perspective of disparity, however, is expanded to include geography, socioeconomics, ethnicity, gender, age, health insurance, and ability, and these are more practical disparity domains for much of SSA (Table 2).

**Table 2:** Major demographic disparity domains in trauma care identified for sub-Saharan Africa

Disparity domain^a^	Description	Rationale
Geography	Subregional, country, province, district and urban–rural divides	Variation between and within 46 distinct nations within the world's fastest urbanizing region with GDP concentrated on productivity of urban centers^b^
Race, tribe, ethnicity and cultural preferences	Tribal differences, majority-minority ethnicities, racial divides, cultural preferences in seeking care	Africa hosts over 3,000 ethnicities and even more languages. Many regions and countries have cultural minorities
Health insurance and social protection	Level of financial risk, and presence or absence of health insurance is an established determinant of health care access	Sub-Saharan Africa had among the widest range of universal health coverage effective coverage performances^c^
Gender	Gender differences in health and the use of health services	Widespread social and cultural factors affect gender dynamics across SSA
Age	The natural vulnerability of adolescents and children coupled with the higher burden of lifelong disabilities and early mortality from injuries	Almost half of Africa’s current population is under 18, and steady growth in births and declining mortality rates will bring Africa’s child population to 1 billion by 2055^d^

^a^Demographic factors on the basis of which disparities can be defined

^b^Urbanization in sub-Saharan Africa. Meeting Challenges by Bridging Stakeholders https://www.csis.org/analysis/urbanization-sub-saharan-africa

^c^https://sph.umich.edu/pursuit/2021posts/future-of-universal-health-coverage-in-africa.html

^d^UNICEF, AU. Children in Africa. Key statistics on child survival and population

## Methods

**Fig. 1:**
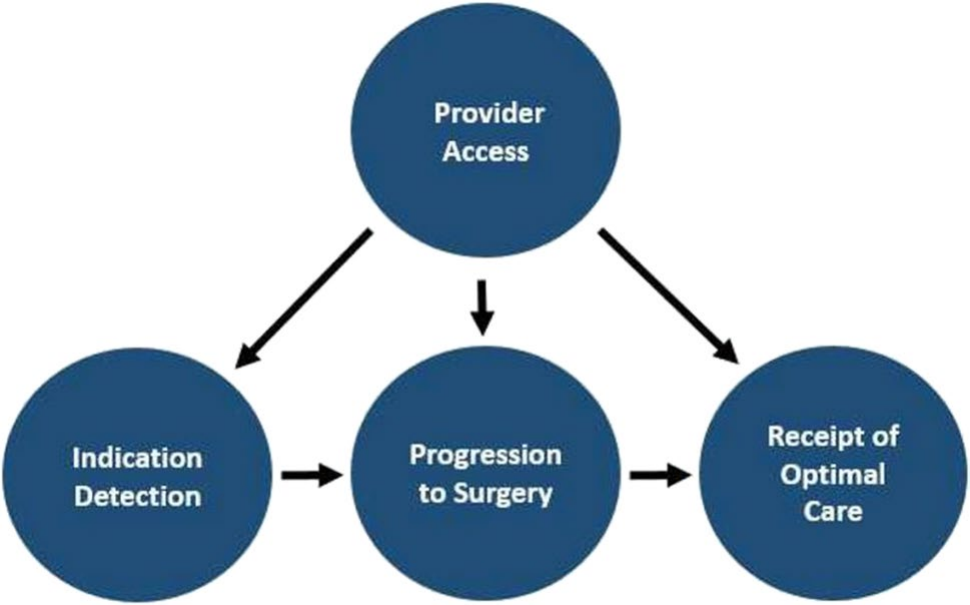
A conceptual model for classifying measures of disparity in trauma care access [16••]. A conceptual model for classifying surgical access disparity measures in the USA. Reprinted from Journal of the American College of Surgeons, Mar;228(3), de Jager E, Levine AA, Udyavar NR, Burstin HR, Bhulani N, Hoyt DB, Ko CY, Weissman JS, Britt LD, Haider AH, Maggard-Gibbons MA., Disparities in Surgical Access: A Systematic Literature Review, Conceptual Model, and Evidence Map., Pages No. 276–298, Copyright (2019), with permission from Elsevier. https://doi.org/10.1016/j.jamcollsurg.2018.12.028

This paper is a narrative literature review of disparities in access to trauma care in SSA utilizing a conceptual model that has been previously described by de Jager et al. [16••] (Fig. 1). The review was informed by a formal literature search in addition to information synthesized from related studies and through disaggregation of data by surgeons in the SSA context.

### Search Strategy and Selection Criteria

#### Search Strategy

PubMed and Embase databases were searched systematically with no language restrictions. Search terms included “Disparity,” “Inequity,” “Trauma,” “Injury,” and “Sub-Saharan Africa.” We utilized 155 terms to capture disparities. In the PubMed search, we included the MeSH terms for Wounds and Injuries, Surgical Procedures, Operative, Resuscitation, Anesthesia, and sub-Saharan African countries. The detailed search string for PubMed is provided in Appendix 1. The search was limited to studies published from 1st January 2010 to 17th July 2021 to ensure results were relevant and recent. We also identified relevant grey literature through a customized Google search, target websites, and consulting with members of the authorship team immersed in different SSA contexts.

#### Inclusion and Exclusion Criteria

We included primary quantitative, qualitative, and mixed-method studies, secondary reviews, letters, and conference proceedings with the primary aim of assessing or describing disparities in access to injury care within SSA (between subregions, between countries, and within countries). These studies focused on all population groups in both rural and urban settings. We excluded studies that did not specifically compare epidemiological characteristics of groups with access to disadvantaged populations. We restricted the review to studies carried out in any of the 48 SSA countries with results from either part or all of the country or spanning multiple countries. We excluded studies focused on disparities in injury epidemiology and those that compared SSA with other parts of the world.

### Identification of Studies, Data Extraction, and Analysis

Search results were collated to Covidence after duplicates were removed using Zotero version 5.0.96. Two reviewers (either KOE, GMD, or BA) performed title and abstract screening for each paper. Conflicts were resolved by a more senior reviewer (BA) and by consensus. Full-text screen was then carried out by 2 reviewers (BA, GMB), and disagreements were resolved by consensus. The main reviewer extracted and analyzed data from all articles. Information extracted from the publications included context of the study (countries and year of publication), methodology (design of the study), disparity domain, and outcomes (identified disparity in access to trauma care). Studies were grouped and narrative synthesized utilizing de Jager et al.’s framework for access disparities [16••] (Fig. 1).

In view of the paucity of studies focused explicitly on disparities in trauma care access within SSA, we included additional information from studies that could inform the narrative synthesized from general surgical disparities and disaggregated data where reasonable to highlight disparities from authors’ experiences.

### Disparity in Trauma Access Conceptual Framework

Unidirectional arrows show the system of influence between the concepts. In the trauma care context, provider access = trauma care provider access; indication detection = surgical indication detection; progression to surgery = progression to trauma surgery/intervention; receipt of optimal care = receipt of optimal trauma care.

We utilized an existing four-component framework from a previous systematic review of literature [16••].

1. Measures of disparity in trauma care provider access reflect disparities in access to the highest quality of trauma surgical care and discharge provider facilities.

2. Measures of disparity in the detection of indications for trauma care. These include disparities in the time of injury presentation, diagnosis, or referral of a potential injury.

3. Measures of disparity in progression to trauma surgery involve disparities in the process of attaining a surgical opinion or procedure once a trauma surgical indication has been detected.

4. Measures of disparity in receipt of optimal trauma care reflect disparities in a patient’s ability to receive the highest quality trauma care.

## Search Results

**Fig. 2:**
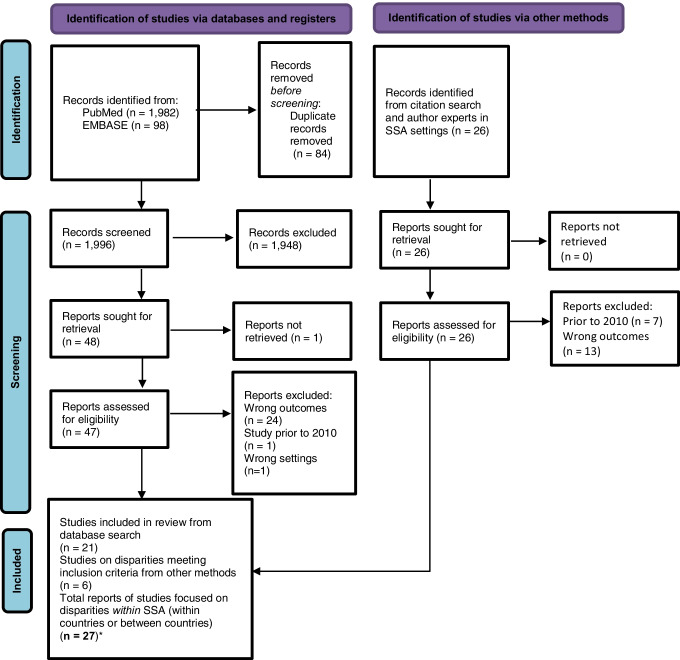
PRISMA flow diagram

The PRISMA flow diagram [21] for the disparities specific literature search (Fig. 2) and 27 included studies (Table 3) are as shown [8, 10, 22–44, 45•, 46].

**Table 3:** Summary of included studies

Study	Country/countries	Scope of disparity	Published year	Study design	Disparity domain	Identified disparity in access to trauma care	Disparity framework
Bearden 2018 [22]	Uganda	Within country	2018	Household Survey	Socioeconomic, geographical	Rural versus urban; untreated surgical (and post traumatic) conditions are significantly more in rural areas than in urban areas	Disparity in trauma care provider access
Bergström 2015 [23]	Mozambique, Tanzania	Between countries	2015	Literature review	Geographical	Rural versus urban; urban retention of surgical care providers is disproportionate and disadvantages rural dwellers	Disparity in trauma care provider access
Butler 2019 [24]	Ghana	Within country	2019	Registry review	Geographical	Rural versus urban; availability of fully trained surgeons is more in urban areas	Disparity in trauma care provider access
Chokotho 2015 [25]	Ethiopia, Kenya, Tanzania, Uganda, Rwanda, Burundi, Mozambique, Malawi, Zimbabwe, and Zambia	Within countries	2015	Web-based survey	Geographical	Rural versus urban; inequitable distribution of accident and emergency units, trauma radiology (C-arm, CT), number of surgeons, anesthetists is unfair to rural dwellers	Disparity in trauma care provider access
Chokotho 2016 [26]	Ethiopia, Kenya, Tanzania, Uganda, Rwanda, Burundi, Mozambique, Malawi, Zimbabwe, and Zambia	Within countries	2016	Web-based survey	Geographical	Rural versus urban; capacity of hospitals in sub-Saharan Africa to manage traumatic injuries is low in rural areas, and better in urban	Disparity in trauma care provider access
Citron 2019 [27]	All sub-saharan Countries	Between countries	2019	Policy review	Geographical	Between countries; absence of trauma care policies	Disparity in receipt of optimal care
Clarke 2014 [28]	South Africa	Within country	2014	Records review	Geographical	Rural versus urban; rural areas as error prone environments	Disparity in receipt of optimal care
Dijkink 2017 [10]	Includes Ghana, Sychelles, South Africa, Zimbabwe	Between countries	2017	Systematic review	Geographical	Only South Africa has a Level II/III prehospital and facility-based trauma care, trauma education and quality assurance	Disparity in receiving optimal care
Edem 2019 [29]	South Africa	Within country	2019	Literature review and Qualitative	Geographical	Rural versus urban; disparities resulting from referral and in -hospital delays to trauma care	Surgical indication detection; progression to trauma surgery
Esquivel 2016 [8]	Zambia	Within country	2016	Geospatial analysis and facility survey	Geographical	Rural versus urban; access to trauma care capable facilities	Disparity in trauma care provider access
Fraser 2020 [30]	Kenya	Within country	2020	Geospatial analysis	Geographical, socioeconomic status	Rural versus urban; poverty resulting in preferential use of public or faith-based facilities with increase travel time by as much as 65%	Disparity in trauma care provider access
Henry 2015 [31]	Malawi	Within country	2015	Hospital administrator and clinician survey	Geographical	Rural versus urban disparity, with no certified surgeons or biomedical technicians in district hospitals- all were clustered at central hospitals	Disparity in trauma care provider access
Juran 2018 [32]	Sub-Saharan Africa	Between countries	2018	Geospatial analysis	Geographical	Between countries, 43% of the population in Central Africa, 33% in Eastern Africa, 40% in Southern Africa, and 34% in West Africa are not within 30 min of a Bellwether procedure/trauma surgery capable hospital. Access was lowest in Eritrea (36%), Angola (41%), Ethiopia, Mauritania (42%), Chad, Madagascar, and Lesotho (46%)	Disparity in trauma care provider access
Kacker 2016 [33]	Cameroon	Within country	2016	Registry review and facility survey	Socioeconomic	Socioeconomic; being in the lowest socioeconomic status quintile was associated with an increased likelihood of having sought initial poor quality trauma care; more wealthy individuals had inequitable access to the trauma center	Disparity in receiving optimal care
Kong 2017 [34]	South Africa	Within country	2017	Retrospective study	Geographical	Rural versus urban; significant difference in mortality between urban and rural patients due to delayed transfers from rural areas	Disparity in trauma care provider access and progression to surgery
Lin 2019 [35]	Uganda	Within country	2019	Geospatial Analysis and facility assessment	Geographical	Rural versus urban; reduced emergency and essential surgical care capability in rural areas	Disparity in trauma care provider access
Ntakiyiruta 2016 [36]	Rwanda	Within country	2016	Geospatial Analysis and retrospective review	Geographical	Rural versus urban; most patients transferred from other provinces	Disparity in trauma care provider access
Ma 2020 [37]	Includes African countries	Between countries	2020	Literature review	Geographical	Between countries; ICU disparities < 1/100,000 except for Southern Africa [South Africa (8.9), Botswana (1.6), Namibia (3.4), and Kenya (1.0)]	Disparity in quality of trauma care
Mould-Millman 2017 [38]	49 of 54 African countries	Between countries	2017	Expert survey	Geographical	Between countries; disproportionately low number of Emergency Medical Systems outside South Africa. Rural versus urban disparity	Disparity in trauma care provider access
Shah 2020 [39]	Cameroon	within country	2020	Financial insurance protection survey	Insurance; socioeconomic	Privately insured versus uninsured; private insurance coverage was a predictor of hospital admission after injury	Disparity in trauma care provider access
Spiegel 2017 [40]	Sierra Leone, Uganda, Mauritania, Benin, Zambia, Burkina Faso, Democratic Republic of Congo and Togo	Between countries	2017	Epidemiologic	Geographical	Between countries; rural versus urban; wide disparities between the countries in the number of facilities per 100,000 population that reported offering basic surgery, comprehensive surgery, and blood transfusion	Disparity in trauma care provider access and progression to surgery and disparity in receiving optimal care
Stephens 2017 [41]	Uganda	Within country	2017	Participatory interviews	Socioeconomic	Socioeconomic; high versus low social capital; (relationships with health care providers, high socioeconomic class) was the greatest predictor of access to surgery	Disparity in progression to surgery
Stewart 2016 [42]	Ghana	Within country	2016	Geospatial analysis	Geographical	Rural versus urban; ill equipped first level hospitals disenfranchise rural dwellers	Disparity in trauma care provider access
Tabiri 2015 [43]	Ghana	Within country	2015	Facility survey and provider interviews	Geographical	Rural versus urban; substantial clinical equipment deficits were found at most primary hospitals disadvantages the rural	Disparity in trauma care provider access
Touray 2018 [44]	The Gambia	Within country	2018	Facility survey	Geographical	Rural versus urban; disparity in availability of intensive care	Disparity in receiving optimal care
World Bank., 2021 [45•]	Botswana, Burkina Faso, Burundi, Cabo Verde, Cameroon, Central African Republic, Chad, Comoros, Congo Brazzaville, Gabon, Lesotho, Liberia, Madagascar, Malawi, Mauritania, Niger, Rwanda, Senegal, Sierra Leone, Sudan, Tanzania, Togo, Uganda, Zambia, and Zimbabwe	Between countries	2021	Emergency Medical Services questionnaire	Geographical; socioeconomic	Between countries; disparity in Emergency Medical Systems policy, type of prehospital care, financing of care, fee for service limits access for the poor	Disparity in trauma care provider access
Yaffee 2012 [46]	Ghana	Within country	2012	Patient survey	Insurance status	Insurance status, uninsured more likely to bypass nearby facilities for injury care	Disparity in trauma care provider access

### Disparities in Trauma Provider Access

#### Disparities in Geographic Access

Two-hour access to a facility that can perform the Bellwether procedures (cesarean section, laparotomy, and open fracture management) has been used as an indicator for access to timely essential surgery [11••]. Some studies have suggested that a relatively high percentage of the SSA population (71–93%) have 2-h access to potential surgery capable facilities at district and tertiary levels using spatially defined travel times [32, 47•]. Regional and district hospitals presumably capable of trauma laparotomies and management of open fracture have been identified and mapped [47•]. Most facilities are government-run, while others are managed by non-governmental organizations or faith-based institutions [32]. In SSA, there are about 0.5 public health facilities per 100,000 population, and for 80% of the population, these are the mainstay of health provision including trauma care [48]. Overall figures, however, may be bloated by small and island countries like Comoros, São Tomé, and Príncipe, who understandably have over 95% of their populations living within 2 h of a surgical health facility. Furthermore, analyses based on geography alone, which is a single component of access, overestimate true access to care [11••, 49, 50]. There are disparities in access between SSA countries. South Africa, Nigeria, and Kenya have greater than 90% access. This falls to less than 50% in Chad, Eritrea, and the Central African Republic. On the other hand, less than 25% of the population of South Sudan has theoretical 2-h access to public hospitals [47•]. This pattern reflects the plight of less industrialized nations with large landmass and the role of conflict in fueling health disparity.

Given the emergency presentation of traumatic injury and the benefits of early presentation, an indicator based on 30 or 60-min facility access may be more appropriate [51–53]. Juran et al. found that 43% of the population in Central Africa, 33% in Eastern Africa, 40% in Southern Africa, and 34% in West Africa are not within 30 min of a Bellwether procedure/trauma surgery capable hospital [32]. Thirty-minute access was lowest in Eritrea (36%), Angola (41%), Ethiopia, Mauritania (42%), Chad, Madagascar, and Lesotho (46%) [32].

Research done within SSA reveals a consistent narrative of marginalization of rural populations [8, 22, 23, 25, 30, 35, 36, 42, 43, 54, 55]. The documented rates of 2-h access might actually largely reflect the widespread urbanization of SSA [32]. Findings from Ghana show that, for most of the population, larger urban hospitals are often the closest facilities with essential surgical care capability [42]. Not only do less than 1 in 5 persons in Somaliland live within 2 h of essential surgery, but most hospitals in Somaliland capable of surgery are within the urban Maroodi Jeex region. Only one hospital was in the more rural regions [54]. In South Africa, for instance, 86% of the population had 2-h access to these facilities, but large, sparsely populated areas of the country did not [55]. Socio-economic factors also play a role in this disparity. For individuals living below the poverty line in Kenya, it has been estimated that preferential use of faith-based facilities or public facilities as is common in much of SSA [56] could increase travel time by as much as 65% [57]. In Ghana, uninsured individuals were more likely to bypass nearby facilities for injury care [46].

Gender-based disparities in surgical care access connected to gender norms, autonomy, decision-making power, and other socio-cultural determinants have been described [58]. However, more research needs to be done on the impact of gender on trauma care access and quality in SSA as little literature is focused on the subject. Malawian women were less likely than men to secure community-sourced healthcare financial aid and more likely to underutilize necessary healthcare [59]. Furthermore, women had delays in time from presentation to operative intervention, undergoing operations later than men for general surgical conditions by an adjusted mean difference of 1.91 days [58]. We found no studies on disparities in access to trauma care access for lesbian, gay, bisexual, transgender, and questioning individuals in sub-Saharan Africa.

#### Disparities in Availability of Emergency Medical Services

“Out-of-Hospital Emergency Care” has been described as a suitable overarching term for use in Africa. It refers to “the full spectrum of emergency care that occurs outside healthcare facilities” and comprises two critical components- first responder care (tier-one) systems and prehospital care and emergency medical services (tier-two) systems. Emergency Medical Service (EMS) systems represent “Formalized prehospital care, provided by emergency care professionals who respond to medical emergencies within a well-defined jurisdiction” [45•, 60].

An expert survey investigating the jurisdiction, operations, resources, and regulation of Emergency Medical Services in 49 of 54 African countries showed the absence of EMS prehospital systems in 33 (61%) [38]. Sixteen countries housed the 25 EMS systems. Only 26% had a toll-free EMS telephone number. Although EMS services exist in a third of African countries, and transport severely injured patients, only 8.7% of the population is within EMS system coverage, and this is significantly skewed towards urban and capital city dwelling individuals. Furthermore, these systems primarily offer only BLS and charge fee-for-service which disenfranchises the poor [38, 45•]. Rural–urban and socio-economic disparities are thus observed in access to EMS, EMS governance and standards, financing, training, and communication standards [38, 45•]. Only three countries in West Africa had confirmed EMS [38]. In this study, South Africa was found to host almost a quarter of EMS systems or agencies, with others identified in Algeria, Botswana, Cameroon, Democratic Republic of Congo, Ethiopia, Ghana, Kenya, Madagascar, Namibia, Nigeria, Sudan, Zambia, and Zimbabwe. Most documented air ambulance services in SSA are concentrated in South Africa [61–63]. In Nigeria, air ambulances are a very expensive endeavor and are run by the military or private organizations and largely limited to operations in the country’s financial and political capitals [64]. Boat ambulances have been documented in the literature for Libya, Nigeria, South Africa, and Rwanda [38, 65].

Despite relatively high growth in EMS coverage from an initial 9% to over 81%, only 5.6% of Ghanaians were able to name the National Ambulance Service and only 3.4% knew the 3-digit public access number [66]. In much of West Africa, ambulances are more associated with carrying bodies than emergency patients [66]. Characteristics of individuals who were disadvantaged by the lack of knowledge in Ghana were unclear, but poor education and cell phone availability may underlie a disparity in access. Rwanda has a robust Service d’Aide Medicale Urgente which coordinates 273 ambulances at the district level, 12 ambulances in Kigali, air ambulances, and a boat ambulance [67]. This contrasts sharply with neighboring Congo which has no formal public prehospital or hospital-based emergency healthcare services [38]. In Nigeria, some success with formal prehospital systems has been recorded in Lagos State and the Federal Capital Territory, the two most highly urbanized States in the Federation. These systems have largely failed elsewhere due to hindrances resulting from cultural, political, and infrastructural implementation determinants [68–70]. Contextualized, informal lay provider networks as employed on a small scale by some parts of Madagascar, Uganda, Ghana, and southern Nigeria [71–75] can be strengthened to decrease prehospital trauma mortality rates; however, this should not be used to excuse the absence of tier-2 systems which ideally represent the highest quality of prehospital surgical care [76]. The percentage of injured patients that arrive at health facilities by ambulance in SSA varies widely by country and facility, from 59% in Ethiopia [77] to 5.9% in Zambia [78] and 5% in Mozambique [79].

#### Use of Low-Volume Hospitals and District Hospitals Versus Trauma Facilities

A 2018 systematic review defined the relationship between high hospital trauma volume and better patient outcomes [80]. In addition, considerable survival advantage has been shown when trauma patients are managed by high-volume surgical teams, independent of hospital volume [81]. Most district hospitals which provide care to rural dwellers are relatively low trauma volume facilities, although much variability exists [82]. Little data is available comparing outcomes in district hospital trauma care with referral center care outcomes, but, intuitively, gaps exist. Despite district hospitals being a mainstay of trauma care in SSA [82] and the high prevalence of traumatic brain injury in the region [83], a scoping review found that very few DHs mentioned capacity to perform burr holes in their essential surgical practice [82, 84]. This is a rural–urban disparity. Low volume rural care providers often have low levels of training and may lack expertise in complex trauma care management [82]. Task shifting and sharing may be a necessity in LMICs; however, task sharers’ must be supervised, and practice volumes must be adequate so as not to disadvantage patients [85].

A study in Nairobi, Kenya found significantly increased delays in emergency presentations when facility choice was restricted to “free” and faith-based facilities. Wait times are often longer, and emergency capacity is many times less adequate in these facilities, yet indigent surgical patients travel 50–60% longer distances to access care at “free” care centers. Individuals living below the poverty line were further away from all facilities than the overall population and were not able to afford nearby private facilities [30].

Many countries in SSA do not have a single dedicated trauma facility. Where they exist, internal disparities in access to these facilities are catalyzed by cost of care, distance from the periphery, and poor ambulance services and filter recipients of care [86]. The Trauma Society of South Africa describes levels I–IV trauma centers as major, urban, community, and primary health facilities. Some bias for geography is implied in this definition, as level II centers are strictly located in urban centers [87]. A rural–urban disparity is seen in the use of low-volume trauma facilities.

#### Disparities in SAO and Trauma Specialist Density

Much of the surgical workforce in SSA is involved with trauma care. Surgical workforce density in the region is about 1.7 per 100,000 population in contrast to 92 and 54 per 100,000 in Europe and North America, respectively [88]. No country has met or exceeded the recommended 20 SAO per 100,000 in SSA [11, 88]. This is far from evenly distributed by country, with Somalia having 0.16 specialists per 100,000 and Burundi having 0.4 per 100,000 in 2018 on one end of the spectrum, while South Africa has over 11 per 100,000 [88]. A study from Sierra Leone found that there were only 2.7 surgical providers/100,000 inhabitants. Non-specialists performed over half of all surgeries. In rural areas, the densities of surgical specialists were 26.8 times lower compared with urban areas overall, and there were four districts without specialists out of 12 [89]. The narrative is consistent across much of Africa. Dahir et al. found that Maroodi Jeex, the capital of Somaliland, had ten surgeons, eight obstetricians, and three anesthesiologists, while rural districts of Sool, Togdheer, and Sanaa had no surgeon, Sahil had no obstetrician, and no anesthesiologist was practicing outside the capital [54]. If surgeons are rare, dedicated trauma specialists and anesthetists are rarer still [90]. In 2014, Ghana had only 24 orthopedic surgeons (0.9 per million) compared to 75.2 per million in the USA, but even these were largely located in urban settings [91]. Inequity in neurotrauma care is even more acute for children, as SSA has 0–1 pediatric neurosurgeons per country except for South Africa [92].

Trauma provider training varies across the continent. A systematic review evaluating emergency medicine training programs in LMICs shows a skew towards East and Southern Africa in literature with more significant gaps in training for West and Central Africa [45•, 93]. Most trauma specialists are wholly or partially trained in South Africa which has robust trauma specialty training [94, 95]. A post fellowship specialty in trauma and surgical critical care was only recently developed by the West African College of Surgeons as a 2-year, full-time program with a 6-month mandatory hands-on rotation at the trauma center of Chris Hani Baragwanath Academic Hospital CHBAH in South Africa [94]. This is currently the only training program for non-orthopedic specialist trauma surgeons in the West Africa region. As of 2021, two fellows were enrolled, and only two general surgeons and one orthopedic surgeon had completed the program [94]. Apart from orthopedic surgeons, other practicing trauma surgeons in West Africa were trained outside the sub-region. This is a good beginning, and this training needs to be scaled up to serve the region’s trauma needs. There is currently no fellowship or post-fellowship specialization offered by the College of Surgeons of Eastern, Central and Southern Africa (COSECSA) in trauma or critical care [96].

Since 2013, COSECSA has partnered with the Royal College of Surgeons of Ireland to execute the Essential Surgical Training program aimed at equipping general practitioners, nurses, and anesthetic technicians in rural hospitals with surgical and referral techniques [97]. Training priority is given to the most rural hospitals [97]. There is no equivalent of this in West Africa, although yearly Trauma Management Courses and Advanced Trauma Operative Management Courses are held in Jos, Nigeria, and Accra, Ghana, respectively [98]. Less than 4 years ago, the National Postgraduate Medical College of Nigeria made Advanced Trauma Life Support mandatory for all surgical specialties at the membership level, in contrast to its adoption in South Africa over 30 years ago [86, 99].

African surgeons often migrate to HICs where there are more opportunities for career advancement, more sophisticated training and practice, better political stability, efficient social and health systems, and higher remuneration for work [100–103], and Africa’s specialist SAOs constitute the highest proportion practicing abroad globally (67·9%) [104]. Trauma provider brain drain occurs to higher income regions as well as within the continent. However, there may be a disparity in surgeon retention between the West African and ECSA regions. Between 1973 and 2013, COSECSA had surgeon retention rates of 85.1% in their country of training, 88.3% in their region of training, and 93.4% in Africa [105]. Naidu et al. also showed that most surgeons who trained at University of Cape Town, South Africa, are still working on the continent [106].

Current rates in West Africa are not yet clearly documented, but anecdotal evidence suggests high rates of migration [107]. Further studies are needed to validate the magnitude of migration of injury management capable SAO providers.

Anesthesiologist density is 16.18 per 100,000 population in South Africa in tremendous contrast to figures of 0–1 in the rest of SSA except for Gabon (1.28) and Namibia (2.44) [108]. This has significant implications for trauma care capacity.

#### Rural Versus Urban Disparities in the Location of SAO Providers

A study from Malawi found no general surgeons, anesthetists, or biomedical technicians in 23 rural district hospitals with a catchment of about five million people as opposed to 27 general surgeons, nine anesthetists, and five biomedical technicians in urban central hospitals with a catchment of two million people [31]. This disparity is even more acute in rural West Africa than the ECSA region as decentralization of training of surgeons (using rural and district hospitals as opposed to urban teaching hospitals alone) has encouraged trainees to stay in ECSA rural areas [105, 109]. The West African College of Surgeons relatively recently introduced a 6-month rural posting earlier in specialist surgical training [110, 111]. The influence of this on rural practice post-specialization is yet to be assessed. Membership diplomas, as an exit level surgical certification by WACS, to increase the mid-level provision of surgical care may also help address the urban confluence of trauma care capable specialists. Chirdan et al. found that 90% of pediatric surgeons (more capable of pediatric trauma care) reside in urban areas and work in tertiary facilities [112].

*The private–public disparity in SAO availability* contributes to disparities in trauma care access. One report described South Africa’s general surgeon density as being comparable to that of the USA if the private sector alone was considered. South Africa has six trained general surgeons per 100,000 insured population working in the private sector. With 60% of specialist surgeons working in private care, only 0.69 per 100,000 population has been left to cater for patronizers of the overwhelmed public health services [113, 114].

*Disparities in advanced surgical training opportunities* may contribute to regional disparities in trauma management capacity. COSECSA is the primary surgical training institution spanning East, Central, and Southern Africa with a presence in 12 countries, 303 accredited trainers in 125 accredited rural and urban hospitals. The WACS is primarily responsible for surgical training in 18 West African countries and covers a region of 381 million people. COSECSA appears to have a wider reach of trainees while most WACS trainees are from Nigeria or Ghana [105, 115–117]. For example, 100% of WACS fellowships, 96% of memberships, and 85% of entry-level trainees in anesthesia for April 2021 were from Nigeria, while 6% of specialists and 15% of entry-level trainees were from Ghana, with none from Francophone West Africa [115–117]. Other than population dynamics, the decentralized COSECSA training model versus the urban-centric, university teaching hospital model of WACS training may contribute to this.

### Disparity in Detection of Indication for Trauma Surgery

This represents disparities in detecting the need for intervention on account of injury and time to specific trauma diagnosis. Little specific data is available from SSA on disparities in time from trauma to detection of indications for trauma surgery; however, delays from the decision to seek care to presentation at a health facility (type 2 delays) and from presentation to definitive injury treatment (type 3 delays) may be a useful proxy for these disparities [118].

#### Delayed Admission, Referral, and Diagnosis

No data was identified comparing countries. In rural Rwanda, almost 50% of patients had a delay in referral. These underscore a rural–urban disparity in attaining final surgical diagnoses [115]. Kong et al. found significant difference in mortality between urban and rural patients due to delayed transfers from rural areas [34]. In Cameroon, possessing private insurance coverage was a predictor of hospital admission after injury. This highlights the disadvantage of the uninsured in getting an appropriate diagnosis [39]. Among patients with traumatic fractures who experienced greater than 24-h delays across 18 LMICs, delays in referral and in the emergency department were found to be the most common (50.5%) [120]. A study in Northern Ghana showed that delays in accessing orthodox trauma care are often due to initial care by traditional bone setters (TBS). The leading perceived advantage of TBS over orthodox care was cheaper fees (75%), suggesting socioeconomic disparities in these delays [121].

### Disparities in Progression to Trauma Surgery

These are disparities in the process of attaining a surgical opinion or procedure for injury once a surgical indication has been detected or of receiving the indicated surgical procedure (decision to treat). A South African study found that delays in receiving care (not in seeking or reaching care) were the largest contributor to avoidable trauma deaths (59%) [29]. Persons for whom surgery was indicated being offered a surgical option for conditions may be delayed because of the lack of a qualified surgical or anesthesia provider, lack of theatre or intensive care unit (ICU) space, inadequate materials for surgery, and lack of insurance or corruption and vested interests of providers seeking to divert patients from public to private trauma care facilities [122–124]. Delays in referral from one level of care to the other and inappropriate transfers are a good marker of this disparity.

In much of SSA, time from incident to definitive management is not systematically recorded, in line with the paucity of trauma registries. From a South African report on penetrating thoracic trauma in an urban center, most patients were in the operating room within 4 h of sustaining their injury (average, 93 min). All patients with 12- to 48-h delays had been observed in peripheral hospitals before being transferred [125]. These findings on urban access contrast with those of a rural South African orthopedic trauma center where 41.4% of presenting patients had been delayed for over 72 h following evaluation at an initial facility [126]. In a report from Malawi of similar traumatic injuries, 26.7% were attended to after 24 h from injury [127]. On the other hand, in a study from Rwanda, the median number of days from injury or first symptom to definitive surgery was 7.3 days [128]. In rural southwest Cameroon, 60.3% of patients needing urgent surgery were delayed for more than 7 days before presentation for definitive care despite the previous contact with healthcare workers [129].

Specific to LMICs, factors like lack of electricity, water, oxygen, or anesthetic gas may delay emergency surgery, and long surgery lists for neglected trauma may cause year-long delays [130]. The paucity of trauma data and non-uniformity within countries as illustrated above makes national comparisons difficult. This variation reflects variation in surgical capacity defined by human resources and equipment [131, 132].

#### Gender-Based Delays in Surgical Care

There is a paucity of data on gender-based disparities in LMIC and SSA trauma care delivery. In Malawi, a study found that women had delays in time from presentation to operative intervention, undergoing operations later than men for general surgical conditions by an adjusted mean difference of 1.91 days [58]. No literature was found assessing gender-based delays in surgical trauma care for the lesbian, gay, bisexual, transgender, queer or questioning, and other populations of patients whose gender identity might differ from their assigned sex at birth.

#### Disparities in Progression to Surgery Based on “Social Capital”

In answering the question, “When surgical resources are severely constrained, who receives care?”, Stephens and colleagues found that patients with advantageous social connections (friends, family, and contacts affiliated with the hospital) were able to self-advocate for orthopedic trauma surgery at a national referral hospital in Uganda. This provides grounds for disparity in progression to surgery [41].

#### Financial-Based Delays in Surgical Care

Even though no studies have identified denial of care to patients without health insurance, a Rwandan study showed that 2% of patients were delayed due to lack of insurance and 5% for other financial reasons [122]. Reports from a qualitative study done in Sierra Leone showed that costs of care at public facilities and the financial strain of needing to pay out-of-pocket interrupted treatment for some injured patients [133]. At the Komfo Anokye Teaching Hospital, Ghana, uninsured children were more likely to experience delays in care for financial reasons than insured children (17.3 versus 6.4%) [134]. In approximately 1 in 10 patients, recommended surgery was refused due to concerns about cost by 9% of patients, among whom two-thirds were uninsured [132]. Delays due to financial limitations can lead to worse health outcomes on top of economic catastrophe [135]. This represents a double tragedy for these households.

Of 1,396 injured patients in Ghana who had indications for surgery at admission, 60% of the uninsured incurred “catastrophic” health costs (more than 10% of their household annual income) as compared with 30% among the insured. As compared with men, women had over double the odds of financial catastrophe from seeking injury care. The researchers, however, found no statistically significant difference in the time to surgery between the two groups nor was the risk of death significantly different [132].

#### Medical Equipment/Infrastructure

Trauma management is dependent on functional equipment. More homogeneous distribution of specialized radiologic resources makes for more equitable trauma care [136, 137]. In East, Central, and Southern Africa, functional mobile C-arm X-ray machines were only available in 4% of district hospitals and 27% of referral facilities. CT scan machines were accessible in only 3% of district facilities and 26% of referral hospitals. Up to 20% of district facilities and 49% of referral facilities reported adequate instruments for the operative fracture management, and only 4–10%, had a sustainable supply of necessary implants [26]. In Zambia, over half of all radiological equipment is in two of ten provinces, serving only one-third of Zambians. Up to 75% of the national equipment inventory is in the public sector, serving 96% of the population [137]. A Ugandan audit found the reverse, with the majority of radiological equipment belonging to private health facilities. Most of the equipment was in the central region which has the third-highest population density [138]. Even within a relatively highly resourced country like South Africa, there were greater than tenfold discrepancies between the least and best-resourced provinces in terms of CT (5.0/million) and MRI (2.9/million) availability [136].

Karekazi and colleagues found that equipment available to neurosurgeons varied across a cohort of specialist neurosurgeons. A CT scanner was available to 86% and MRI to 38%. Over 14% of neurosurgeons had no access to essential equipment [139]. A report from Tanzania in 2020 found that 95% of all severely injured patients needed but did not receive a brain CT scan. A majority (80.8%) of these patients also needed but did not receive oxygen [140]. Up to 60% of neurosurgeons managing pediatric neurotrauma reported needing a drill for pediatric neurosurgery [92].

### Disparities in Receiving Optimal Care

This reflects disparities in a patient’s ability to receive the highest quality surgical care and postoperative follow-up. The Trauma Systems Maturity Index developed by the WHO is useful in detecting trauma system maturity on a spectrum from least mature (level I) to most mature (level IV) [10]. An international overview of trauma systems which included Ghana, Seychelles, South Africa, and Zimbabwe, identified a mature system (level III) only in South Africa. Inadequate trauma planning and lack of data can be responsible for poor quality care and feed these disparities [141, 142]. In addition, Kacker found that patients presenting to the largest trauma hospital in Cameroon were wealthier than the broader community, while being in the lowest socioeconomic quintile was associated with previous poor-quality care, suggesting inequitable access to optimal care [33].

#### Trauma mortality patterns (Trauma Specific Perioperative Mortality Rates)

Perioperative mortality rate (POMR) has been utilized as a global indicator of access to safe, quality surgical care [11]. It refers to death after surgery and anesthesia on the day of surgery, or before discharge, or within 30 days of surgery [143]. Disparities in POMR between conflict and the rest of SSA exist [144, 145]. In addition, disparities in POMR between conflict regions within SSA have been described, and these may reflect variations in surgical skill and post-operative care capacity. Davies et al. observed POMR in conflict areas of South Sudan (Leer and Nasir), Central African Republic (Boguila), and the Democratic Republic of Congo (Mweso, Kimbi, Baraka, and Shamwana). The POMR on days 2 and 30 were significantly higher in interventions for violence-related injuries and in South Sudan compared with DRC [144]. Disparities in optimal pediatric surgical care are also seen in conflict arenas. Being younger than 15 years of age and undergoing laparotomy was significantly associated with death between 2 and 30 days [144]. Though this may reflect childhood malnutrition or more severe presentations in this age group, variation in skills of surgery providers and post-operative care capabilities must be considered. Pediatric patients must be viewed as a particularly vulnerable group in SSA conflict settings [144].

#### Disparity in the Availability of Intensive Care Units and Blood Banks

One review found no data for acute care beds in SSA [146]. However, ICU bed capacity, of renewed interest in the COVID-19 pandemic, is generally < 1/100,000 except for Southern Africa ICU oasis of South Africa (8.9), Botswana (1.6), Namibia (3.4), and Kenya (1.0) [36]. Even within countries, ICU availability is skewed to urban locations [44]. This is both a measure of disparity in trauma care capacity and pandemic preparedness [147].

Blood availability in SSA is severely limited [148]. A modelling study based on the WHO Global Status Report on Blood Availability suggests that in the region, only South Africa has sufficient blood to meet local transfusion needs (inclusive of injury care) [148]. The largest relative gap between demand and supply was found in South Sudan, where only 46 units were supplied to meet the need of 3,537 per 100,000 people resulting in a 75 times greater demand than supply [148]. Within countries, a key disparity is that most blood donation centers and banks are in urban centers, inaccessible to those in rural areas [40, 149, 150].

#### Disparity Between Quality of Adult and Pediatric Trauma Care

Ranging from road traffic-related blunt trauma, to falls from trees, gunshots, burns, and ingestion of glass in several reports [151–153], the diversity of trauma etiology for children and adolescents in SSA is heightened by their vulnerability and dependence. For instance, at outset of injury, up to 38.2% of children in Northern Ghana were taken to traditional bonesetters rather than hospital [153]. There are widespread reports of neglect of children and adolescents in trauma care [112, 151, 154, 155]. Pediatric trauma is peculiar, as children are unique in anatomical proportions, physiology, and risk and constitute a wide spectrum of ages. SSA poses a challenge for children [156], not only because of the trauma risk but also because of disparities in access to pediatric surgeons and pediatric trauma facilities. Burundi had no pediatric surgeon in 2016 and a co-author from that context suggest that there is currently still no pediatric surgeon serving the country [157]. Nigeria had a historical geographical disparity of pediatric surgeons with most of the limited number located in the more affluent south (1 surgeon to 1.5 million children) as compared to the north (1 surgeon to 3.3 million children) [157]. No pediatric trauma specialist training programs exist in West Africa. The availability of these training programs in SSA might be limited to South Africa. Physician non-specialists, general surgeons with only 3 months of trauma or pediatric surgical training [158], and non-physician healthcare providers can only cater adequately to patients with minor to moderately severe injuries. Exposure to pediatric surgery by undergraduates is both limited and varied [159]. There is a real need, more outside of South Africa, for trained pediatric trauma surgeons to manage severely injured children.

#### Rehabilitation Facilities for Trauma

In SSA, 210 million people or one in five individuals experience conditions that could benefit from rehabilitation (including trauma) [160]. Rehabilitation goes beyond physical therapy, and an acute shortage of psychiatrists in SSA contributes significantly to this narrative [161]. The highest rehabilitation need is in Nigeria where 41 million people could potentially benefit; however, only 250 psychiatrists, mostly located in urban centers, serve 200 million people [161]. In a survey of hospitals spanning Zimbabwe, Zambia, Uganda, Tanzania, Rwanda, Mozambique, Malawi, Kenya, Ethiopia, and Burundi, only 58.5% of all referral hospitals had rehabilitation units. This dipped to 35.7% for district hospitals [26]. A 2011 study of a tertiary institution Ghana showed that of 84 patients with rehabilitation needs, no inpatients got needed acute inpatient rehabilitation, only 14% got physical therapy (even then once a day for less than a week), 0% had occupational therapy, and 18% received any form of assistive device as part of trauma treatments. This shortage of rehabilitation is heightened in rural settings. Over half of all Anglophone countries in SSA do not have physiotherapy or occupational therapy programs and face an acute shortage. As of 2017, South Africa and Nigeria were the only countries offering masters and doctoral-level training in physiotherapy and occupational therapy [162].

## Future Directions and Recommendations

### Identifying and Filling Gaps in Knowledge About Trauma Care Disparities

More research specific to trauma care disparities needs to be done in SSA. Key future directions of this research should focus on identifying current gaps in knowledge and deficiencies in trauma care training in medical and nursing schools. A formal scoping review by the authors to explore the extent to which geography, gender, socio-economic, tribal, and age-related disparities are considered in trauma care research from Africa is in view. Further research should also aim to fill these gaps with new knowledge in the areas like disparities in the availability of blood banking services for trauma, availability of acute trauma care, postoperative follow-up care for trauma patients, and COVID-related disparities specific to trauma care. Future studies need to address disparities in trauma access for ethnic minorities within countries in West, Central, and East Africa.

### The Role of National Trauma Registries in Defining Disparities

Inequality monitoring is “the process of generating evidence on how various subpopulations within a country are performing regarding health, in order to inform equity-oriented policies, programs and practices that ensure that disadvantaged or hard-to-reach populations are not left behind. This relies on the collection, analysis and reporting of health data disaggregated by inequality dimensions, such as sex, age, economic status, education, place of residence, ethnicity and other context-specific population subgroups. In this light, health information systems are the foundation for monitoring health inequality” [12]. Trauma registries often document presentation, care, and outcomes of care for injured and hospitalized patients. Information from these registries is used to improve the efficiency, quality of trauma care, and permit regional comparisons [163]. Until more recently, many sub-Saharan countries did not have accurate and robust trauma data, and this hinders surgical planning, evidence-based policy, and monitoring [141, 164]. Currently, few countries in SSA have national trauma registries. South Africa, Malawi, Uganda, Cameroon, Kenya, Ghana, Rwanda, Tanzania, and Zambia [168] have established trauma registries. Botswana [165], Nigeria [164], Ethiopia [166], and Mozambique [79, 167] have registries at different levels of implementation. Quality of data collection is a challenge in many countries [169]. Uganda and South Africa’s Injury Surveillance Systems serve as examples of the benefits of such injury and mortality measurement systems implemented in a context-specific manner.

Access to the highest quality trauma care can only be possible in the presence of continued quality improvement [79, 164–168]. The absence of trauma registries feeds disparities in the quality of trauma care by denying trauma systems information that can focus efforts on parts of the system in greatest need of improvement.

### Addressing Disparities Through the Implementation of National Surgical Plans

**Fig. 3:**
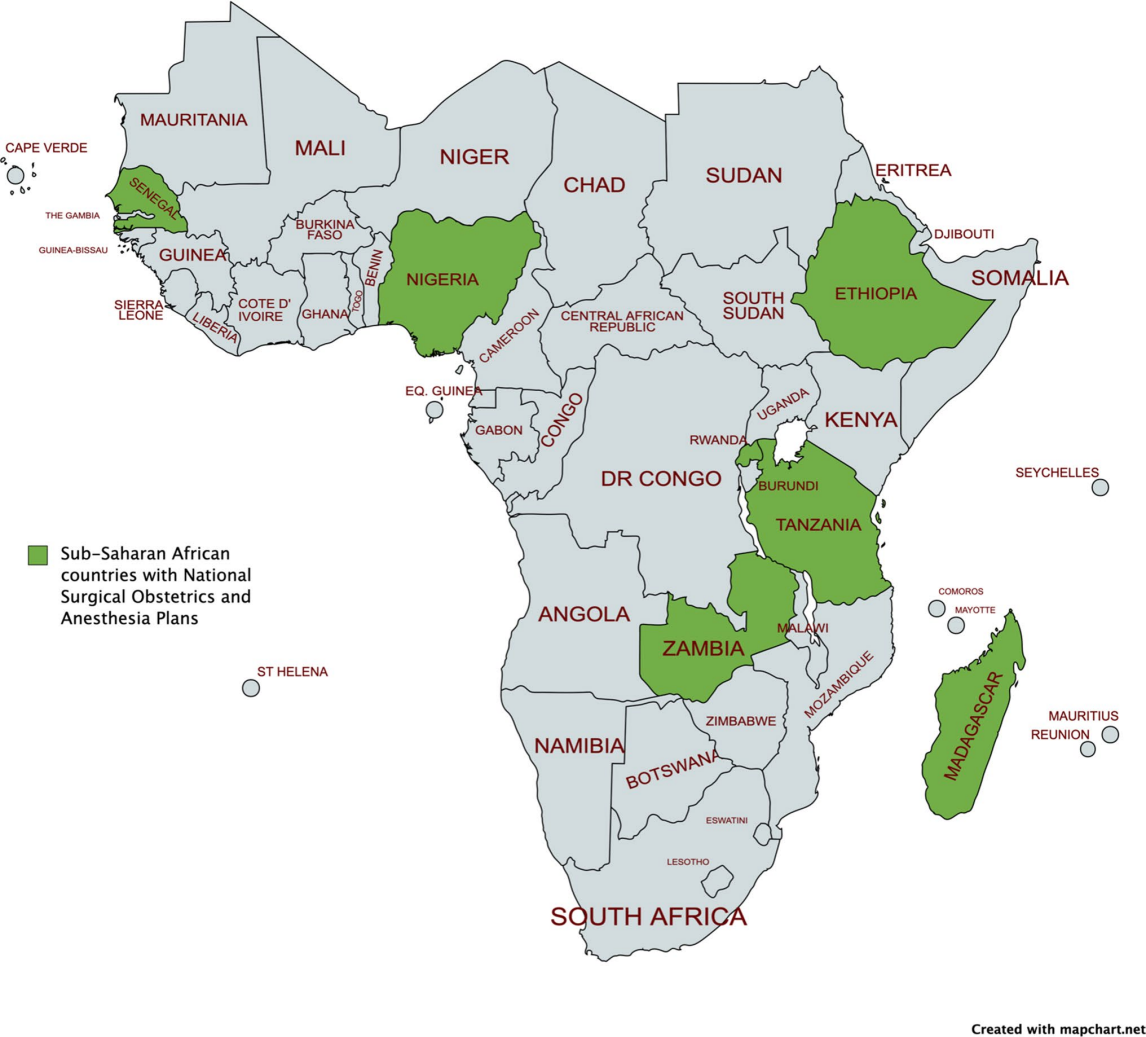
Completed National Surgical Obstetrics and Anesthesia Plans across SSA

Historically, surgical and trauma care planning has been neglected on policy levels [27]. For instance, the word “surgery” is mentioned in South Africa’s Department of Health’s National Strategic Plan 2015/2016–2019/2020 only once (referring to cataract surgery) [170]. National Surgical Obstetrics and Anesthesia planning is an important policy movement born after the Lancet Commission on Global Surgery, which is focused on systematically strengthening surgical systems in domains of infrastructure, service delivery, surgical workforce, information management, financing, and governance as part of a country’s national health plan. Trauma systems can potentially be strengthened by planning, and though nomenclature may differ, the process is essential. Beginning with Senegal, some SSA countries including Zambia, Nigeria, Ethiopia, Madagascar, Rwanda, and Tanzania have developed deliberate National Surgical Obstetrics and Anesthesia Plans (NSOAPs) (see Fig. 3) [27, 142, 171]. As an example, the Nigerian National Surgical Obstetrics, Anesthesia, and Nursing Plan contains sections deliberately focused on trauma care delivery and has the potential to promote equitable access to trauma management [172].

Work is ongoing in all regions, notably in the Southern African Development Community and Francophone West Africa to develop NSOAPs, and the state of affairs is dynamic. However, currently, National Surgical Obstetrics and Anesthesia Plans (NSOAPs) seem to have taken root in East Africa (36% of African Union region countries), but notably, Central Africa (0%) is lagging in adopting and implementing the process. In the entire West African region, only 2 countries, Senegal and Nigeria (13%), have developed NSOAPs, while 10% of Southern African countries have NSOAPs [171]. Senegal commenced in advance of the Lancet Commission on Global Surgery; however, other Francophone countries have not yet launched NSOAPs. These gaps have the potential to manifest in the future as disparities in trauma access. However, efforts are underway in several countries to develop a holistic surgical plan [171]

### Frameworks for Addressing Disparities in SSA

Addressing these disparities can only be done by addressing trauma “staff, stuff, space, and systems” using a holistic framework. The WHO health systems framework can be directly applied to trauma care in SSA with evaluation and strengthening of country level and regional trauma service delivery, the trauma workforce, health information systems, trauma related medical products and technologies, trauma care financing, and leadership and governance [173]. Emphasizing strategic trauma policy frameworks like the World Health Assembly resolution on trauma and emergency care services (resolution WHA 60.22) [174, 175] and regional injury care coalition building will be key. A better understanding of these disparities will involve regional and country-level assessments and partnerships. These assessments cannot be unilateral or purely academic but will be multilateral and include academic institutions, clinical personnel, public-health experts, non-governmental organizations, global surgery advocates, policymakers, private organizations, and government institutions. Coalitions like the Bellagio Essential Surgery Group have modelled team contributions to reducing disparities in access to trauma care, and their recommendations should be considered by SSA stakeholders (Table 4) [176••]. The WHO Global Alliance for Care of the Injured with its focus on trauma systems development, evidence and research, trauma registries and data, continuing education and capacity building, and advocacy is another example of the potential of partnerships, accompaniment, and action in identifying and addressing injury care disparities [177]. Ranging from using simple tools like the WHO Trauma Care Checklist [178] to applying complex policy documents like NSOAPs, implementers should utilize a wide range of synchronized approaches to eliminating disparities in trauma care. No uniform approach will work for all SSA in addressing social determinants of health. Contextualized universal health coverage for injury that truly protects from catastrophic expenditure must be developed, as high insurance coverage does not necessarily result in equity in access to trauma care. Training of trauma management capable health providers and leveraging on non-specialist physicians, while improving regional trauma specialist training in local institutions with local resources is essential. Governments must plan and implement trauma care policy and not pay lip service to injury care funding. Human, systemic and historical factors supporting these disparities including implicit and explicit bias, must be clearly identified and addressed.

**Table 4:** Bellagio group recommendations [176••]

Recommendations
Recommendation 1: Strengthen surgical services at district hospital levels
Recommendation 2: Improve systems for trauma care delivery
Recommendation 3: Expand supply and quality of health workers with surgical capabilities
Recommendation 4: Build evidence to inform interventions to improve access to surgery in sub-Saharan Africa

## Comment on the “Why" Behind Global Disparity in Access to Surgical Care

The underlying causes of access disparities are hydra-headed and not easily generalizable. However, poverty fertilizes disparities. Poverty in Africa is due to both implicit and explicit bias, and there are larger historic, political, moral, and structural factors at play [179]. No discussion on disparities in SSA would be complete without acknowledgment of Africa’s history of slave trade, colonialism, neo-colonialism, and apartheid, and its effect on trauma care access along racial lines [180]. SSA inherited a system of oppression, perpetuated by greed, pride, and white supremacy. Years of European colonial rule were largely disproportionate to colonialist investment in local health care and worse still surgical care. The motivation and focus of most European medical services in Africa was to provide curative and preventive care for colonizers, settlers, troops, and leadership. Only when ill-health of indigenous populations threatened the well-being of the expatriate population did these structures focus on their care. Little emphasis was placed on building enduring indigenous medical educational institutions after dismantling traditional ones. Post-colonial structural adjustment programs, inequitable resource extraction, and the set-up of global trade, information, and economies which destroys local agriculture and manufacturing also contribute to this narrative. This thread weaves through to current poverty in Africa, challenges of governance, and high unemployment despite rich resources. More recently, neocolonialist surgical structures have focused on short-term reconstructive missions without a system approach—a type of hit and run model many times focused on diseases that are not the greatest of local needs [180]. Neo-colonialist self-contained surgical platforms that are convenient for the global north have undermined local surgical system strengthening. Neo-colonial humanitarian aid and inequitable partnerships that do not instill indigenous responsibility or require local leadership have perpetuated these disparities. Unfortunately, these are the foundations on which Africa’s medical, surgical, and trauma care were built [180].

These historical disparities are highlighted by documented events like the 1977 post-injury death of Steve Biko, founder of the anti-apartheid Black Consciousness Movement, completely unattended on the floor of an empty cell. Resulting from racial profiling, implicit and explicit bias, repeated medical negligence, physician complicity, and inequity in the use of ambulance services involving a 1,100-km (12 h) journey to care in the back of a police van without medical escort for a severely head-injured patient, South Africa lost one of its foremost political thinkers to trauma [181]. At the end of the social experiment of apartheid in 1994, healthcare reform was placed high on the country’s development agenda. Despite this prioritization and marked improvements in equity, health outcomes remain “polarised, unequal and unfair” with black and rural South Africans as vulnerable subgroups [182]. This untenable situation must never be normalized.

## Limitations

This review has been limited by the lack of available trauma-specific data. Thus, relevant general disparities in surgical care have been included. The search of databases showed that relatively little research has been done directly addressing disparities in trauma care in SSA, and so much of our information has been synthesized from surgical disparities through disaggregation of data. Of the 48 SSA countries, no specific data was available on internal disparities on all domains for more than half: a data disparity in and of itself.

The possibility of publication bias was increased by our narrative approach, as the methodological quality of the selected articles was not assessed; however, our research question did not lend itself to a systematic review, and a scoping review would be more focused on analyzing the wide gaps in the literature. This would not have afforded the authors the opportunity to develop a more aggregate view of the subject.

Furthermore, search literature on which we based this review spanned about 10 years. Some countries would have improved their trauma systems since the publication of some of these articles. Unpublished improvements could not be addressed in this review; however, authors working in various countries gave context. Despite reviewers carrying out a multi-lingual search, this review may also have been anglophone-country focused. More contexts are needed from Francophone sub-Saharan Africa.

## Conclusion

South Africa with its long history of trauma associations, trauma conferences, and injury legislation hosts the highest number of trauma centers and the largest number and spread of ambulance services; however, functional prehospital trauma care still favors only the well insured, and there is no national registry. In contrast, most other SSA countries have underdeveloped trauma systems. Inequity in access can be reduced by prehospital initiatives, as used in Ghana, and community-based insurance, as modelled by Rwanda. Across SSA, the largest geographical gaps in trauma care are presumably in central Africa, francophone West Africa, and conflict regions of East Africa.

Consistent in the SSA narrative is the rural–urban disparity in trauma care access and the disadvantage of the poor. On a continent where much of the population is in the pediatric age group, significant disparities also exist between adult and pediatric trauma care. Most research focused on disparities has emerged from South Africa probably because of its highly racialized society and history of apartheid coupled with its advanced trauma care capacities. Even in the absence of racial issues, many other SSA nations need to critically assess and document equity in access to trauma care. Recognition of these disparities should drive creative equitable solutions and focused interventions, partnerships, accompaniment, and action.

## Supplementary Information

Below is the link to the electronic supplementary material.

Supplementary file1 (DOCX 13.3 KB)

## Data Availability

The authors confirm that the data supporting the findings of this study are available within the article [and/or] its supplementary materials.
