# Supplementary material for: Disparities in Access to Trauma Care in Sub-Saharan Africa: a Narrative Review
Source: Curr Trauma Rep. 2022 Jun 6;8(3):66–94. doi: 10.1007/s40719-022-00229-1 (PMC9168359; doi:10.1007/s40719-022-00229-1)
Supplement: Supplementary file 1 — Supplementary file1 (DOCX 13.3 KB) [file 40719_2022_229_MOESM1_ESM.docx]

Appendix 1

Search Strings

PubMed

((ethnic disparities[TIAB] OR ethnic disparity[TIAB] OR health disparities[TIAB] OR health disparity[TIAB] OR healthcare disparities[MH] OR health care disparities[TIAB] OR healthcare disparities[TIAB] OR health-care disparities[TIAB] OR health care disparity[TIAB] OR healthcare disparity[TIAB] OR health-care disparity[TIAB] OR health status disparities[MH] OR (disparities in health[TIAB]) OR J Health Care Poor Underserved[Journal] OR J Health Dispar Res Pract[Journal] OR J Racial Ethn Health Disparities[Journal]) OR (culturally competent care[MH] OR culturally competent care[TIAB] OR delivery of health care[MH:noexp] OR disparities[TIAB] OR health behavior[MH] OR health behavior[TIAB] OR health behaviors[TIAB] OR health inequality[TIAB] OR health inequalities[TIAB] OR health inequities[TIAB] OR health inequity[TIAB] OR health knowledge, attitudes, practice[MH] OR health related quality of life[TIAB] OR health-related quality of life[TIAB] OR health services accessibility[MH] OR health services, indigenous[MH] OR health services needs and demand[MH] OR health status disparities[MH] OR mass screening[MH] OR mass screening[TIAB] OR mass screenings[TIAB] OR patient acceptance of health care[MH] OR patient selection[MH] OR quality of health care[MeSH Major Topic:noexp] OR social class[MH] OR social class[TIAB] OR social determinants of health[MH] OR social determinants of health[TIAB] OR social disparities[TIAB] OR social disparity[TIAB] OR social factors[TIAB] OR social inequities[TIAB] OR social inequity[TIAB] OR socioeconomic factor[TIAB] OR socioeconomic factors[MH] OR socioeconomic factors[TIAB] OR socioeconomically disadvantaged[TIAB]) AND (African ancestry[TIAB] OR african continental ancestry group[MH] OR ageism[MH] OR apartheid[MH] OR Asian[TIAB] OR asian continental ancestry group[MH] OR Asians[TIAB] OR White African[TIAB] OR White Africans[TIAB] OR Caucasian[TIAB] OR Caucasians[TIAB] OR disabled[TIAB] OR disabled persons[MH] OR disabled persons[TIAB] OR diverse population[TIAB] OR diverse populations[TIAB] OR emigrants and immigrants[MH] OR ethnic group[TIAB] OR ethnic groups[MH] OR ethnic groups[TIAB] OR ethnic inequalities[TIAB] OR ethnic population[TIAB] OR ethnic populations[TIAB] OR ghetto[TIAB] OR ghettos[TIAB] OR health services for persons with disabilities[MH] OR homeless[TIAB] OR homeless persons[MH] OR immigrant[TIAB] OR immigrants[TIAB] OR inmate[TIAB] OR inmates[TIAB] OR jail[TIAB] OR jail population[TIAB] OR jail populations[TIAB] OR medically underserved area[MH] OR medically uninsured[MH] OR minorities' health[TIAB] OR minority group[TIAB] OR minority groups[MH] OR minority groups[TIAB] OR minority health[MH] OR minority health[TIAB] OR minority population[TIAB] OR minority populations[TIAB] OR migrant worker[TIAB] OR migrant workers[TIAB] OR Native Africans [TIAB] OR Native African [TIAB] OR people of color[TIAB] OR poverty[MH] OR poverty[TIAB] OR poverty areas[MH] OR poverty area[TIAB] OR poverty areas[TIAB] OR prisoner[TIAB] OR prisoners[MH] OR prisoners[TIAB] OR race factors[MH] OR race factors[TIAB] OR race and ethnicity[TIAB] OR racial and ethnic minorities[TIAB] OR racial discrimination[TIAB] OR racial disparities[TIAB] OR racial disparity[TIAB] OR racial equality[TIAB] OR racial equity[TIAB] OR racial inequities[TIAB] OR racial inequity[TIAB] OR racial prejudice[TIAB] OR racial segregation[TIAB] OR racism[MH] OR refugees[MH] OR refugees[TIAB] OR rural health[MH] OR rural health[TIAB] OR rural health services[MH] OR rural population[MH] OR rural population[TIAB] OR rural populations[TIAB] OR sexism[MH] OR slum[TIAB] OR slums[TIAB] OR social discrimination[MH] OR social marginalization[MH] OR social segregation[MH] OR transients and migrants[MH] OR underserved[TIAB] OR undocumented immigrants[MH] OR medically uninsured[MH] OR uninsured[TIAB] OR urban health[MH] OR urban health services[MH] OR urban population[MH] OR urban population[TIAB] OR urban populations[TIAB] OR vulnerable population[TIAB] OR vulnerable populations[MH] OR vulnerable populations[TIAB] OR working poor[MH] OR working poor[TIAB])) AND (("wounds and Injuries"[Mesh] OR Trauma*[tiab] OR "injuries"[sh] OR injury[tiab] OR injuries[tiab] OR burn[tiab] OR burns[tiab] OR fracture*[tiab])) OR ((Trauma) AND ("Surgical Procedures, Operative"[Mesh] OR "surgery"[sh] OR "Resuscitation"[Mesh] OR "Anesthesia"[Mesh] OR surgery[tiab] OR surgical[tiab] OR operative[tiab] OR perioperative[tiab] OR postoperative[tiab] OR preoperative[tiab] OR resuscitation*[tiab])) AND ("africa south of the sahara"[MeSH Terms] OR ("africa"[All Fields] AND "south"[All Fields] AND "sahara"[All Fields]) OR "africa south of the sahara"[All Fields] OR ("sub"[All Fields] AND "saharan"[All Fields] AND "africa"[All Fields]) OR "sub Saharan africa"[All Fields] OR angola*[tiab] OR benin*[tiab] OR botswana*[tiab] OR burkin*[tiab] OR burundi*[tiab] OR cabo verde*[tiab] OR cape verde*[tiab] OR cameroon*[tiab] OR central africa*[tiab] OR chad*[tiab] OR congo*[tiab] OR côte d'ivoire[tiab] OR ivorian*[tiab] OR ivory coast[tiab] OR guinea*[tiab] OR eritrea*[tiab] OR eswatini*[tiab] OR ethiopia*[tiab] OR gabon*[tiab] OR gambia*[tiab] OR ghana*[tiab] OR kenya*[tiab] OR lesotho[tiab] OR liberia*[tiab] OR madagascar*[tiab] OR malawi*[tiab] OR mali[tiab] OR malian[tiab] OR malians[tiab] OR mauritania*[tiab] OR mozambi*[tiab] OR namibia*[tiab] OR niger[tiab] OR nigerien*[tiab] OR nigeria*[tiab] OR rwanda*[tiab] OR são tomé and principe[tiab] OR senegal*[tiab] OR sierra leone*[tiab] OR south africa*[tiab] OR tanzania*[tiab] OR togo[tiab] OR togolese*[tiab] OR uganda*[tiab] OR zimbabwe*[tiab])

Embase

('disparities'/exp OR 'disparities' OR 'disparity'/exp OR 'disparity') AND ('injury'/exp OR 'injury') AND ('africa south of the sahara'/exp OR 'africa south of the sahara')
